# Prognostic Factors for Delayed Healing of Complex Wounds in Adults: A Scoping Review Protocol

**DOI:** 10.3390/nursrep12040087

**Published:** 2022-11-23

**Authors:** Raquel Marques, Marcos Lopes, Paulo Ramos, João Neves Amado, Paulo Alves

**Affiliations:** 1 Centre for Interdisciplinary Research in Health, Institute of Health Sciences, Universidade Católica Portuguesa, 4169-005 Porto, Portugal; 2 School of Nursing Department, Universidade Federal Ceará, Fortaleza 60020-181, Brazil; 3 Unidade de Saúde Familiar Corino de Andrade, 4490-602 Porto, Portugal; 4 School of Nursing Department, Universidade Católica Portuguesa, 4169-005 Porto, Portugal

**Keywords:** chronic wound, decision making, prognosis, treatment, wounds and injuries

## Abstract

(1) Background: The high prevalence of persons with wounds and its consequences for a person’s quality of life makes the issue a relevant focus of attention for healthcare professionals. Through prognostic factors for healing, the individual risk of complications can be predicted, is possible to predict imminent delays and guide decision-making, thus helping healthcare professionals. (2) Methods: A scoping review performed according to JBI methodology and guided by the Preferred Reporting Items for Systematic Reviews extension for Scoping Reviews (PRISMA-ScR) checklist will aim to identify the studies that meet predefined eligibility criteria. Five databases and gray literature will be the sources used to research adults with pressure ulcers, venous leg ulcers, arterial ulcers, or diabetic foot ulcers and report the prognostic factors for delayed healing in any care setting. (3) Results: This review will consider all quantitative and mixed studies in the last five years. The selection of articles will be carried out by two reviewers independently, using EndNoteWeb and Rayyan. Prognostic factors will be presented by design study, sampling, setting, outcome, wound type, and statistical methods. (4) Conclusions: Mapping prognostic factors for delayed healing could also be a starting point for a systematic review and meta-analyses to quantify the value of each factor.

## 1. Introduction

It is estimated that there are around 1.5 to 2 million persons living with a chronic or complex wound, some of them for a period longer than six months [1]. As an example, in the United Kingdom (UK) 1.47 per 1000 persons have a chronic wound [2]. The presence of a wound that is hard to heal compromises the health-related quality of life in general [3,4]. The costs inherent to the treatment are substantial; for example, diabetic foot ulcers (DFUs), venous leg ulcers (VLUs), and pressure ulcers/injuries (PUs/PIs) cost 5056.71, 7886.05, and 5972.28 GBP per person, respectively, and over GBP 4 billion between 2017 and 2018 to the UK National Health Service [5].

There is no consensus on the best term to describe superficial or deep tissue injuries that are difficult to resolve with standard treatment due to the presence of one or more factors that delay healing [6]. These wounds can be called chronic, stagnant, stalled, or hard to heal. We chose the complex wound because it reflects the dynamic and multifactorial healing process and not just the prolonged healing time [7,8,9]. These wounds are common in the elderly population with various underlying diseases, such as diabetes, peripheral venous disease, and peripheral arterial disease, or in a dependent condition with reduced mobility [6]. Complex wounds differ from acute wounds—those that follow their normal healing process—by the presence of several factors that delay healing. The factors that delay healing cause a decrease in mitogenic activity and an increase in the inflammatory response and oxidative stress, resulting in a stagnant and unregulated inflammatory phase with the presence of senescent cells; consequently, they do not progress to tissue repair [6]. For example, PUs/PIs, leg ulcers (venous, arterial, mixed, lymphatic, or combined), DFUs (neuropathic, ischemic, or mixed), malignant wounds, atypical wounds (Marjolin ulcers, pyoderma gangrenosum, calciphylaxis, scleroderma, and sickle cells), and complicated acute wounds, among others, can be considered complex wounds [10].

Today, on a global scale, people live longer while affected by diseases, and people manage to live with more diseases or health problems than in the past; therefore, it is important to develop prognostic studies [11]. Research on prognostic factors aims to understand and improve future results in persons with a specific health condition [11]. In the specific case, persons with complex wounds are known to report that complete wound healing is the most important outcome for them [12]. The healthcare practitioner can, therefore, individualize interventions with objectives that are changeable when they have early knowledge of the severity of the wound, the likelihood of delayed healing, and the patient’s anticipated outcomes.

A preliminary search in February 2022 was carried out in MEDLINE, Cochrane Reviews, and JBI evidence synthesis, and a scoping review was found in which the objective was to obtain information on what factors may have potential prognostic value for the delayed healing of various types of non-traumatic skin ulcers [13]. However, this review only included studies that were published in databases; the search was conducted until 2017; and its findings presented prognostic factors for healing. It is important to update the current scoping review to include more types of leg ulcers, research sources, and map the factors related only to delay, as no systematic reviews were found that gave continuity to the prior review. Thus, this scoping review will aim to identify prognostic factors for the delayed healing of complex wound types in adults.

## 2. Materials and Methods

The proposed review will be conducted in accordance with the JBI methodology for scoping reviews [14,15] and will be guided by the Preferred Reporting Items for Systematic Reviews extension for Scoping Reviews checklist (PRISMA-ScR) [16]. This review protocol was registered on the platform Open Science Framework (osf.io/59xyb).

### 2.1. Inclusion Criteria

To answer the review question “What prognostic factors delay the healing of complex wound types in adults?”, the below inclusion criteria will be considered.

#### 2.1.1. Participants

This review will consider studies that include adult persons aged 18 or over chronologically with complex, chronic, stalled, stopped, or hard-to-heal wound(s).

For complex wounds, we will include DFUs (Wound, Ischemia, and foot Infection (WIfI) classification grade I or higher, or another classification system), VLUs (C6 of Clinical-Etiology-Anatomy-Pathophysiology (CEAP) classification or other classification system that considers wound interruption in the skin barrier), lower extremity arterial disease (LEAD) with critical limb-threatening ischemia (CLTI) formerly designated as Critical Limb Ischemia [17] (in an open wound, Rutherford classification 5 to 6, or Fontaine classification IV), PUs/PIs (category 2 or higher of Pressure Injury Staging System), that do not heal, delay healing, or do not reduce in size (length, width, area, depth, volume, or perimeter in cm or mm) by 20 to 50% in four weeks or 30 days.

We consider wounds healed when the area is equal to 0 cm^2^, 0 mm^2^, or complete epithelialization and wounds in the process of healing when there is a reduction in size in four weeks.

#### 2.1.2. Concept

This review will consider studies that explore prognostic factors for delayed healing. The prognostic factors included are all associated with the attributes of the patient, the wound characteristics, clinical indicators, and socio-economic status. Those associated with the effect of the specific dressings, and studies with a commercial proposal and/or comparison among treatments will be excluded. Thus, we will consider only prognostic factors, that is, those independent of treatment.

A prognostic factor(s) may be examined as a continuous or categorical variable, and any cut-off or dichotomizing/categorizing approach will be included.

We will include prognostic factors where the estimate independently contributes to predicting the outcome and a relationship between exposure and outcome is established. We will not include inconclusive studies, only with a proven effect for delayed healing, and we will consider statistical significance when *p* < 0.05.

Studies of prognostic models will also be included, provided that they report separate associations of individual prognostic factors with delayed healing.

We consider delay time to be equal to or greater than four weeks because if the wound does not reduce by 20–50% (<50% for DFUs, <40% for VLUs, and 20–40% for PUs) in size with appropriate treatment [18,19,20,21] in this period, it will hardly heal without a more specific intervention [20]. A recent study with machine learning models was developed using data from electronic health records to predict patients at risk for non-healing wounds and found no significant differences regarding healing time at four, eight and 12 weeks [22]; based on this study, we decided to use a period equal or greater than four weeks.

#### 2.1.3. Context

This review will consider studies in any context of care (e.g., hospital, community, home, and institutions) provided by healthcare professionals.

We consider the healthcare professionals in the care of the person with a wound to be physicians, nurses, and podiatrists.

#### 2.1.4. Types of Sources

This scoping review will consider quantitative and mixed studies. Quantitative designs include any experimental study designs (including randomized controlled trials, non-randomized controlled trials, or prognostic studies based on data from randomized controlled trials) and observational studies (prospective and retrospective cohort studies). Guidelines issued by national and international wound and tissue viability associations will also be included, as will dissertations or theses published in repositories. Texts and opinion articles, case studies, systematic and narrative reviews, letters to the editors, and in vitro and animal studies will be excluded.

### 2.2. Search Strategy

A three-step search strategy is recommended [14]. An initial search was carried out on MEDLINE to locate articles relevant to the review and to analyze whether they could contribute to the increase in keywords and search terms (Table A1). The second search will be more complete in the databases included with all keywords and indexing terms. Finally, a final reading of the references of the included studies to identify any studies that may have been missed will be conducted.

The search strategy will aim to locate both published and unpublished studies and/or papers.

The search for published studies will be performed using the following databases: MEDLINE via PubMed, CINAHL and Nursing & Allied Health Database via EBSCOHost, Scopus, Cochrane Library, and Web of Science.

The gray literature search will include the following: RCAAP—Open Access Scientific Repository of Portugal; ProQuest—Theses and Dissertations; CAPES theses database; RENATES—National Register of Theses and Dissertations; Online Dissertation Abstracts (EThOS); Google Scholar; and national and international wound and tissue viability reference associations (European Wound Management Association, European Pressure Ulcer Advisory Panel, Association for the Advancement of Wound Care, Wound Source, Sociedad Iberolatinoamericana Úlceras y Heridas, Brazilian Society of Wound Nursing and Aesthetics, Wounds Canada, Australian Wound Management Association, Associação Portuguesa de Tratamento de Feridas, and ELCOS (Sociedade Portuguesa de Feridas)).

The search will initially include studies available and recorded online within the last five years. This time limit is due to the last review having been carried out until 2017, not including any study from this year; it is also due to the numerous publications and revolutionary advances in the area, which have evolved evolution and updated the scope of action with people with wounds. For the complete reading of the articles, those written in English, Portuguese, Spanish, and French, with ethical approval and complete, will be selected. Articles not available in full in the databases could be located through the university library, or the authors could be contacted.

### 2.3. Study Selection

The selection of studies will be carried out by two reviewers independently, and the decision to include the final articles will be discussed with the research team. The selection of relevant studies in relation to the review question will be performed using the flowchart following the indications of PRISMA-ScR [16], demonstrating the process from the initial research to the final selection of studies for extraction and synthesis, including how many articles will have been included or excluded at each step.

All results obtained through the search strategy will be transferred to EndNote Web software (Clarivate Analytics, PA, USA), through which duplicates will be removed. For the reading of titles and abstracts independently by the two reviewers, we will use Rayyan Qatar Computing Research Institute platform.

### 2.4. Data Extraction

Data will be extracted from papers included in the scoping review by two independent reviewers using a data extraction tool developed by the reviewers based on the previous study [13]. The data extracted will include specific details about the following: scoping review details; eligibility criteria; author, year, and country; setting; participants; sampling type and size; wound details (including wound type, grade/severity, and classification system, if applicable); follow-up or cohort time; average healing time; outcome; prognostic factors by wound type; statistical methods; limitations mentioned by the authors; and level of evidence according to JBI classification [23]. A draft extraction tool is here provided (Table A2). Disagreements will be resolved via discussion in the team. Some authors of the articles will be contacted to request missing or additional data. Considering the objective of this scoping review, the quality of the articles will not be systematically evaluated using critical appraisal tools; however, a discussion will be held among the reviewers about the quality of the studies.

### 2.5. Data Analysis and Presentation

To identify, characterize, and summarize the main results, tables will be presented with the main prognostic factors by type of complex wound according to the characteristics of the person, wound and clinical indicators, number of publications per year and country, study design, average healing time, statistical methods, and level of evidence according to the JBI classification.

## 3. Discussion

A scoping review protocol is important because it establishes the objectives, methods, and reporting of the review in advance and ensures the transparency of the process. The protocol should list the criteria that the reviewers intend to use for including and excluding sources of evidence, as well as for identifying relevant data, and how data is to be extracted and presented. The protocol includes the plan for the review and is important to limit the occurrence of reporting bias. Thus, an a priori protocol should be established before performing a scoping review to produce a well-conducted review [14].

Numerous difficulties arise from the complexity of wounds, making it difficult to provide the patient with good diagnosis and therapy [7]. To ensure evidence-informed decision-making and effective treatment for the person with a wound, the healthcare professional must equip himself with different resources and knowledge. Thus, knowledge of prognostic factors can predict individual risk of complications and is possible to warn of imminent delays and guide the professional’s conduct [11].

In order to promote quicker and more effective healing, there are a number of elements that can affect wound healing, making the procedure more complicated, drawn out, and/or accompanied by abnormal tissue repair. The known prognostic factors that influence the healing of complex wounds can be divided into those directly related to the wound, and local and systemic ones related to the general condition of the person, with local factors being the best predictors [24]. The local factors described are wound types [24,25,26], size (area and volume) [22,24,25,26,27,28,29], stage/category [28], number of wounds [25,28], duration of wound [27,28,29], initial wound shape [25], anatomical location [22,24,25,27], hypoperfusion [30], and treatment [25,27]. The factors related to the person commonly referred to are age [25,31], sex [25], days of hospitalization [22,28], and associated pathologies such as renal failure, diabetes, peripheral arterial disease, heart failure, malnutrition, inflammatory diseases, and autoimmune diseases [25,26,27,28]. However, we consider it necessary to map and update the dispersed knowledge about the prognostic factors responsible for delay by the type of the most frequent complex wounds, the methodologies used by the included studies, and the statistical methods addressed, and this review could be the starting point for a systematic review. Prognostic factors can be used to successfully predict healing and may play an increasing role in determining wound severity and treatment [6].

This review will be important to improve the knowledge of wound healing, as it will provide important guidance for clinical practice and the management of patient expectations [32].

## 4. Conclusions

For a well-conducted review, a priori guidelines should be established before conducting the scoping review. This protocol will guide a scoping review that may provide valuable information for understanding the delay in the healing of some of the most frequent complex wounds. Mapping prognostic factors for delayed healing could also be a starting point for a systematic review and meta-analyses to quantify the value of each factor.

## Data Availability

Not applicable.

## Appendix A

**Table A1:** Search strategy used in one of the databases—MEDLINE (via PubMed)—in March 2022.

Search:	Medline via Pubmed (14 March 2022)	Records Retrieved
#1	“prediction”[Title/Abstract] OR “predictions”[Title/Abstract] OR “predictors”[Title/Abstract] OR “predictable”[Title/Abstract] OR “predict”[Title/Abstract] OR “predicts”[Title/Abstract] OR “predicting”[Title/Abstract] OR “predictive”[Title/Abstract] OR “predicted”[Title/Abstract] OR “predictability”[Title/Abstract] OR “prognostication”[Title/Abstract] OR “prognoses”[Title/Abstract] OR “prognosis”[Title/Abstract] OR “prognostic”[Title/Abstract] OR (“prognostic”[Title/Abstract] AND (“criteria”[Title/Abstract] OR “score”[Title/Abstract] OR “characteristics”[Title/Abstract] OR “factor”[Title/Abstract] OR “indicator”[Title/Abstract] OR “biomarker”[Title/Abstract] OR “determinant”[Title/Abstract] OR “decision”[Title/Abstract] OR “algorithm”[Title/Abstract] OR “outcome”[Title/Abstract] OR “risk”[Title/Abstract] OR “variable”[Title/Abstract]))	2,256,950
#2	“healed”[Title/Abstract] OR “healing”[Title/Abstract] OR “heal”[Title/Abstract] OR “healings”[Title/Abstract] OR “heals”[Title/Abstract] OR “cicatrical”[Title/Abstract] OR “cicatrix”[Title/Abstract] OR “cicatrization”[Title/Abstract] OR “cicatrize”[Title/Abstract] OR “cicatrized”[Title/Abstract] OR “cicatrizing”[Title/Abstract] OR “cure”[Title/Abstract] OR “restore”[Title/Abstract] OR “restored”[Title/Abstract] OR “restores”[Title/Abstract] OR “restoring”[Title/Abstract] OR “skin over”[Title/Abstract] OR “repairability”[Title/Abstract] OR “repairable”[Title/Abstract] OR “repaire”[Title/Abstract] OR “repaired”[Title/Abstract] OR “repair”[Title/Abstract] OR “repairing”[Title/Abstract] OR “repairs”[Title/Abstract] OR “regenerating”[Title/Abstract] OR “regeneration”[Title/Abstract]	1,021,613
#3	“chronic wound*”[Title/Abstract] OR “complex wound”[Title/Abstract] OR “wound”[Title/Abstract] OR “wounds”[Title/Abstract] OR “non-healing wound”[Title/Abstract] OR “healing impaired wound”[Title/Abstract] OR “persistent wound”[Title/Abstract] OR “slow healing wound”[Title/Abstract] OR “foot ulcer”[Title/Abstract] OR “leg ulcer”[Title/Abstract] OR “pressure ulcer”[Title/Abstract] OR “pressure injury”[Title/Abstract] OR “pressure injuries”[Title/Abstract] OR “diabetic foot”[Title/Abstract]	250,584
#4	“young adult”[Title/Abstract] OR “adult”[MeSH Terms] OR “adult”[Title/Abstract] OR “adults”[Title/Abstract] OR “middle aged aged”[Title/Abstract] OR “middle aged”[Title/Abstract] OR “aged”[MeSH Terms] OR “aged”[Title/Abstract] OR “80 and over”[Title/Abstract]	8,647,923
#5	#1 AND # 2 AND #3 AND #4	2724
#6	#1 AND # 2 AND #3 AND #4 AND (y_5[Filter])	1148

## Appendix B

**Table A2:** Data extraction instrument.

**Scoping Review Details**	
Scoping Review title:	
Review objective/s:	
Review question/s:	
**Inclusion/Exclusion Criteria**	
Population	
Prognostic factors by wound types	
Context	
Types of evidence source	
**Evidence Source Details and Characteristics**	
Citation details	
Country	
Setting	
Participants (details)	
**Details/Results Extracted from Source of Evidence**	
Sample type and size	
Wound details, including wound type, grade/severity, and classification system, if applicable	
Follow-up time	
Average healing time	
Outcomes	
Prognostic factors by wound types	
Statistical methods	
Limitations mentioned by the authors	
Level of evidence according to JBI classification	
